# Centering social-technical relations in studying platform urbanism: intersectionality for just futures in European cities

**DOI:** 10.1186/s42854-021-00027-z

**Published:** 2021-11-08

**Authors:** Natasha A. Webster, Qian Zhang

**Affiliations:** 1 Present Address: Department of Human Geography, Stockholm University, 10691 Stockholm, Sweden; 2 Department of Social and Economic Geography, Uppsala University, Uppsala, Sweden

**Keywords:** Platform urbanism, Intersectionality, Gig economy, Migrant, Work, Urban inequalities

## Abstract

Platform-based services are rapidly transforming urban work, lives and spaces around the world. The rise of platforms dependent on largely expendable labour relations, with significant migrant involvement, must be seen as connected, and as replicating larger social processes rather than merely technological changes. This perspective paper urgently calls for an intersectional perspective to better understand social-technical relations crossing the digital-urban interface of platform urbanism in contemporary European cities. Critics of platforms and gig work, to date, have mainly focused on algorithms-based social control, degraded working conditions, problematic employment relations and precariousness of gig work. The ongoing Covid-19 pandemic has both disrupted and amplified these issues, intensifying the vulnerability of gig workers. For example, in Sweden, migrant groups and gig workers were separately identified as being hardest hit by Covid, but with little attention to the interconnectivity between these categories, nor to how these groups are co-positioned vis-a-vis larger socio-economic inequalities. Thus, we argue for a deeper understanding of the social processes underlying platforms and for active investigation of how inequalities are being produced and/or maintained in/by these processes. Urban planners, designers and policy makers will need to actively address the hybrid (digital and physical) urban spaces produced in platform urbanism in order to prevent spatial and economic inequalities. We argue for a stronger recognition of interrelated and overlapping social categories such as gender and migrant status as central to the construction of mutually constitutive systems of oppression and discrimination produced in and through the platform urbanism.

## Policy and practice recommendations

- Intersectionality centers social-technical relations in understanding the (re) making of cities by platform urbanism
- Recognition of the production of hybrid (physical and digital) urban spaces is needed in urban planning and policy
- Policy makers need to understand the range and diversity of urban platforms/gig economies
- Migrants and vulnerable workers need to be protected in all stages and spaces of platform urbanism
- All actors, including consumers, involved in platforms need to be included in policy and initiatives

## Science highlights

- Platform urbanism may replicate and maintain social divisions and inequalities
- Technocratic or exceptionalism approaches mask key socio-economic processes and inequalities
- Intersectional approaches may help identify power relations associated with platform urbanism
- Policy makers must understand social relations in gigs and platforms to prevent further inequalities

The twenty-first century has seen an unprecedented rise in accessible advanced technologies; driven by the Covid-19 pandemic, digital platforms and apps have been further normalized, entrenched and embedded into urban life through household services, social connectivity and various forms of personal care; driving a ‘technological everyday’ (Barns, 2019), forming new configurations of living and working through ‘platform urbanism’(Barns, 2020; Leszczynski, 2020; van der Graaf & Ballon, 2019). Concomitantly, recent decades have given rise to inequalities in European cities in the form of social, economic and residential segregation (Andersson et al., 2018; Cassiers & Kesteloot, 2012; Haandrikman et al., 2021). Some migrant communities are locked-out of conventional labour markets while at the same time are increasingly spatially segregated. The rise of platforms dependent on largely dispensable labour relations, heavily involving migrants (van Doorn et al., 2020), must be seen as connected, and as replicating these social processes. The new practices and spatial forms generated by the diverse and volatile components of platform urbanism (Stehlin et al., 2020), negotiate the digital-urban interface in context-specific ways (Odendaal, 2021), to reconfigure the public-private, and state-society relationships (Leszczynski, 2020) and reshape political possibilities for addressing existing social relations (Fields et al., 2020). Future city design, work and leisure hinge on the ways technology/data are governed (Graham, 2020; Sadowski, 2020) but it is not enough to unpack black boxes of algorithms for developing urban solutions (Fields et al., 2020; Wood et al., 2019), nor is it sufficient to allow technocratic, commercial and top-down perspectives to dominate policy formulation and discourse, identification of initiatives, and imaginaries of the urban futures (Odendaal, 2021; Söderström et al., 2014).

We argue that platform urbanism is the remaking of urban geographies, mediated and negotiated through digital technologies, which may replicate and maintain larger social divisions while driving significant structural and systemic changes towards working and social life. One particularly illustrative area of this argument is the connection between platforms, labour and consumption relations; the so-called gig economy. Definitions of the gig economy are varied but should include a) the use of digital technologies and platforms and b) the reordering of labour relations. The gig economy as part of platform urbanism reorders labour relations and reconfigures consumption relations in cities. Through the matching and intermediation of web-based platforms and location-based apps, an increasing proportion of the working population are doing various gig (one-off) jobs from micro-tasks to consultant-like assignments (Berg et al., 2018). While the gig economy has been hailed for providing new forms of working models, including those for small business, findings on this have been mixed (Fudge, 2017; Webster & Zhang, 2020). Migrant gig workers are undoubtedly vulnerable (van Doorn et al., 2020) and tend to be pushed into more precarious, lower paid and less skilled jobs, facing more social and economic precarity (Berglund et al., 2021; van Doorn, 2017; Vyas, 2020). Nevertheless, gig work, especially at the platform level, remains positioned as creative business models, meeting contemporary consumer preferences, especially during pandemics, etc. As platform urbanism becomes more established, the challenge is to improve outcomes, especially through protection of vulnerable workers, including addressing poor working conditions, through sound regulation and policies (Kaine & Josserand, 2019). While policy-based interventions are necessary, there is a need to address the larger core challenge: gig and platform economies are based on the replication and maintenance of social divisions. Altering the latter will require increased awareness of the workings of platform urbanism, and ultimately significant value change.

Accordingly, we argue for a deeper understanding of the power domains underlying platform urbanism and for investigation of how social inequalities are being produced and/or maintained by these processes. While studies and definitions of gig work have focused on how the work is performed or implemented (Aloisi, 2016), highlighting the tensions between worker flexibility and insecurity (de Stefano, 2016), and the ambiguity of labour relations (Minter, 2017), most studies have shied away from the more uncomfortable questions of power: who is delivering and who is consuming these services, and at the urban scale, the question of how to seriously consider urban transformation while probing the embedded inequalities central to these business models. Understanding the role and impact of platforms on the socio-economic and physical fabrics of cities is a theoretical and empirical challenge, not least because many platform activities occur in private spheres (i.e. homes and companies) and in hybrid (digital and physical) spaces. The practicalities of researching platforms mean many processes are obscured in algorithms and private databases, despite the visibility of some stages of gig work, like deliveries (Fields et al., 2020). Notwithstanding, urban planners and policy makers will need to address the hybrid digital-urban spaces produced by platform urbanism in order to increase urban well-being, including prevention of spatial and economic inequalities. Researchers need to shift their thinking about the platform and gig economy from seeing the reordering of working relations as exceptional economic practices to recognizing platform urbanism as a socio-economic process whereby inequality is dynamically ordered, shaped through collective actions and a complicit maintenance of social divisions that shape the winners and losers of the city. Thus, we argue for the need of an intersectional perspective, a stronger recognition of interrelated and overlapping social categories such as gender, racialization, class and migrant status, as central to a better understanding of oppression and discrimination produced in and through the platform urbanism. To ‘open the black box’ of platforms demands deeper engagement with politics and power, and creative methods (Datta & Odendaal, 2019; Fields et al., 2020), for analyzing the political, social, economic and geographical conjunctures that condition the algorithms harm (Safransky, 2020), while going beyond explaining “digital mediations of capture, dispossession, and adverse incorporation” (Elwood, 2020, p. 209).

Intersectionality is a groundbreaking theoretical approach in social sciences but has yet to make significant strides in understanding inequalities in studies of platforms and gig work. Arising from Black feminist thought (Cooper, 2015; Hancock, 2016), intersectionality is an approach to explore the relationships between socially ascribed meanings and values to social categories such as racialization, gender and/or class, to individual and collective experiences (Crenshaw, 1991; Valentine, 2007). Intersectionality highlights the interlocking relationships of power and oppression from a multi-axis framework. This emphasis on complexity offers theoretical insights into the ways hybrid digital-urban spaces are interwoven from and through social divisions (Elwood, 2020). It also helps clarify the ways systems of oppression and advantage are produced together and are dependent on the maintenance of these systems. One of the key critiques and challenges to the approach has been identifying how intersectionality can be translated into policy. Intersectionality does offer potential for policy as the approach shifts thinking from singular explanations/solutions to recognizing the roles and experiences of differently situated populations (Hankivsky & Jordan-Zachery, 2019). Intersectionality in policy can also prevent reductionism by creating space for longer timelines in social and economic issues, encouraging multiple and diverse methods and blurring the boundaries between the recipients of policy targets and non-beneficiaries (Manuel, 2006). Intersectionality centers the diversity of human experiences, thereby potentially improving resource and response development, addressing overlapping policy gaps, and identifying co-produced or concurrent forms of inequalities among other benefits.

As platform urbanism produces complex social-technical relations simultaneously in intersecting physical and digital geographies, possibilities for exacerbated inequalities and opportunities for just interventions co-exist. The latter requires the type of nuance afforded by intersectional perspectives, and has much to offer policy makers. From an intersectional perspective, groups producing and consuming through the gig economy can be positioned as different power domains; but all as agents in creating a system of living and working that is rapidly changing urban lives. Platforms intermediate between consumers and producers, but these relations are not neutral or passive throughout the transaction nor in the hybrid digital-urban spaces produced. The scale and impact of hybrid digital-urban geographies and gig work became immediately apparent early in the Covid-19 crisis. In the Swedish example of a soft lock-down context, we see clearly how platform urbanism is experienced differently by a consumer, a gig worker, or company management. Gig workers play a significant, but uneasy, role in maintaining/producing the operation of supply systems and our daily work and lives by delivering services to households, also within the health, the elder- and homecare sectors. Gig work has likely contributed, but little studied yet, to the exacerbated inequalities through the interactions between the Covid-19 pandemic, work forms and existing divides by migrant and socioeconomic statuses (Drefahl et al., 2020). As an illustration of this challenge, the Swedish health authorities early on identified the links between work and risk of Covid-19 infection with taxi drivers and cleaners among the highest risk and “other service workers”, likely many gig workers, fell within the top six riskiest types of work (Folkhälsomyndigheten, 2020a). Concomitantly, migrant women from Somalia and Ethiopia in Sweden, often discriminated in the standard labour market, was one of the hardest hit groups (Folkhälsomyndigheten, 2020b). Moreover, healthcare centers with many irregularly employed and hourly wage earners, positions often held by migrants and women, were more affected (Fjällborg, 2020). Migrant groups and precarious workers were separately identified as heavily impacted by the Covid-19 pandemic but the ‘silo’ed presentation of these findings would be more efficacious with an intersectional analysis of the interlocking inequalities of work forms and differently situated populations.

As the above example suggests, an intersectional approach would highlight how work and social injustice are inter-connected. Intersectional understandings would provide evidence for better policy and urban planning; from an urban governance point of view, lifting these power inequalities and centering social-technical relations would create opportunities for policy interventions and creative urban solutions such as bringing together the co-production of inequalities and migrant labour. Moreover, this perspective brings spaces of the digital platform to the fore as a socio-economic space through which individuals move and build social environments as much as in the brick and mortar city. The challenge for researchers and policy makers seeking to understand platform urbanism is to take on the difficult task of identifying the intersections of social inequalities embedded in gig and platform work, apart from platform infrastructure and technology, and connect these to effective urban policy. If we wish to fully understand how cities are being reshaped, redefined and rebuilt through platform production and consumption, a bird’s eye view and a bottom-up, context-sensitive approach are simultaneously needed -platforms do not occur outside of social relations, insulated by technology. We need to both understand the network of activities, digital and non-digital, embedded in platforms. We must not fall into the trap of seeing the platform as something different or existing outside of interlocking power relations because of the private and digital character of these processes. Algorithms will tell us some of the story, but we must engage with the larger processes and implications of urban dynamics and change. Only by centering people working in the platform economy, identifying why they are participating in gig work, and how they experience platform urbanism, can we begin to build secure, fair, and just work, and challenge planners, policy makers and technology companies to create fair and just cities.

## Data Availability

Not applicable.
